# 

**Published:** 1892-08

**Authors:** 


					﻿Whole Number,
CCCLXXI.
New Series.
VOL. XXXHI.
No.
^August, 1892.
Buffalo
Medical 3 Surgical
Journal.
Established 1845 by AUSTIN FLINT, M. D.
Editors:
THOS. LOTHROP, M. D.
WM. WARREN POTTER, M. D.
Associate Editors:
WILLIAM C. KRAUSS, M. D.
JOHN A. MILLER, Ph. D.
JAMES WRIGHT PUTNAM, M. D.
DeLANCEY ROCHESTER, M. D.
JOHN PARMENTER, M. D.
J
>«
o
in
ci
CO
l—l
$
H
X
b
O
X
o
>
Q
<
Z
Q
IM
<
cu
fa
•—i
o
o
ci
CO
M
O
t—i
X
CL
z
o
b
&
>—<
o
co
X
o
0
0
LU
I
CO
J
m
D
1
£
0
z
d
I
r
■<
European Agency,
TRUBNER & CO.,
.57 and 59 Luogate Hill, London, E. C.
BUFFALO:
1892.
Foreign
Subscription, $2.50
Entered at the Post-Office at Buffalo, N. Y., as Second-class Mail Matter.
Times Printing House, Buffalo, N. Y,
Table of Contents on Page V.
				

